# The meaning of health to persons aging with longstanding multiple sclerosis

**DOI:** 10.1002/nur.22409

**Published:** 2024-07-02

**Authors:** Alexa K. Stuifbergen, Heather Becker, Nani Kim

**Affiliations:** The University of Texas at Austin School of Nursing, Austin, Texas, USA

**Keywords:** aging, definition of health, multiple sclerosis

## Abstract

Understanding how persons aging with a chronic condition view their health is essential for planning and delivering person-centered care. The purpose of this study was to explore how persons aging with the chronic and disabling condition multiple sclerosis (MS) describe their health and how this has changed over time using data from Years 1 to 26 of an ongoing longitudinal survey study of health promotion and quality of life for persons with MS. The survey included measures of perceived meaning of health, self-rated health, health behaviors and quality of life outcomes. The sample included 168 persons with MS who returned the survey at Time 1 (1996) and again at Time 26 (2022). In 2022, participants had a mean age of 70.13 (*SD* = 8.19) and had been diagnosed with MS for an average of 34.47 years (*SD* = 6.27). Sixty percent of participants rated their overall health as good or excellent. Decreases in health self-ratings over time were not significant. Participants consistently agreed more strongly with items reflecting a wellness-oriented view of health than those reflecting a more clinical/biomedical model. At both time points, clinical definitions of health were negatively related and wellness definitions were positively related to reported frequency of health behaviors. Findings suggest that persons aging with the chronic condition of MS may be more responsive to health messages that emphasize function in daily living, carrying out normal responsibilities, and adjusting to changes in environment and demands. Patient or Public Contribution: Persons with MS provided study data, input on design, and construct measurement.

## 1 | INTRODUCTION

During the last decades, health care for those living with chronic and disabling conditions has expanded beyond symptom and disease management to include broader efforts to promote health and quality of life. For persons living with the chronic disabling condition of multiple sclerosis (MS), evidence suggests that engaging in a variety of health behaviors may improve functional limitations, disease course, and quality of life outcomes (Becker et al., 2022; Flores et al., 2023; Stuifbergen et al., 2016; Wallack et al., 2016). Health promoting behaviors are generally self-initiated, continue over time, and based on the individual’s value and meaning of health. This is quite different than disease and symptom management efforts that are driven largely by health care providers with their training and values often couched in the biomedical model of health. Unlike disease management, health promotion efforts must involve and be driven by the values of the person/patient—values which may be different than that of the clinician (Pender et al., 2015).

If we are to be successful at helping clients/patients promote their health, we need a better understanding of how consumers view and define their health, especially in the context of living with chronic conditions and the experience of aging (Gessert et al., 2015). Definitions and meanings of health are value-based, multidimensional and differ across contexts and, and vary over the life span (Gessert et al., 2015; Van Druten et al., 2022). Change in perceptions and meaning of health over time have rarely been explored and may be critical for people aging with long-term chronic conditions. The primary purpose of this study was to determine how persons aging with a chronic and disabling condition (MS) describe the meaning of health for themselves and how these meanings have changed over a 26-year period. In addition, we explored how meaning of health and self-rated health status were related to health behaviors, and quality of life outcomes.

## 2 | BACKGROUND

Discussions about the meaning of health are not new and can be seen in the writings of ancient Greeks (Bradley et al., 2018). Most recent discussions and explorations are anchored in the 1948 World Health Organization (WHO) definition of health as a “state of complete physical, mental and social well-being and not merely the absence of disease or infirmity” (Kelley, 2008). Given the inclusions of the word “complete” in this WHO definition, persons with chronic and disabling conditions could not be considered “healthy” (Van Druten et al., 2022). While this definition is rarely used today, its influence in both education and practice is still reflected in conceptualizations of health that rest in the biomedical paradigm.

Van Druten and colleagues (2022) conducted a scoping review of 75 articles (published post 2009) focused on conceptualizations of health in different contexts (e.g., patients vs. health care providers, persons with chronic conditions, persons aging). Their findings, based on a wide range of qualitative and quantitative studies, suggest there is no consensus on an overall conceptualization of health that would replace the dated WHO definition. Their analysis noted similarities across study findings including health as multi-dimensional and subjective and not merely complete well-being or functioning as suggested in the biomedical model. This finding is consistent with the notion that in all societies, different views on health, illness, preventive and treatment approaches coexist (Bautista-Valarezo et al., 2020).

Van Druten and colleagues documented important differences in how health care professionals and consumers/patients view health. Complete well-being or functioning was mentioned often by care providers (a biomedical view similar to the WHO definition) but rarely mentioned by patients. Consumers tended to value self-management in their descriptions, while health care providers did not focus on self-management in their conceptions of health. “Patients” in general focused on daily functioning while older adults focused on participation.

Krahn and colleagues (2021) suggested a new definition of health that incorporates the perspectives of living with a disability or chronic condition: “Health is the dynamic balance of physical, mental, social, and existential well-being in adapting to the conditions of life and the environment” (p1). Using this definition, health is distinct from function and thus good health can exist in the presence of limitations associated with chronic illness, disability, or aging. The definition acknowledges the contextualization of individual meanings of health in life experiences and highlights the importance of environmental and social factors on health (Krahn et al., 2021).

Whitmore and colleagues (2023) used qualitative methods to explore factors influencing self-reported health among older adults (*N* = 15, average age 73.6 years) residing in the community. Although most had been diagnosed with at least one chronic condition, 73% rated their health as good, very good, or excellent. Four key themes were identified: (1) Health as a personal responsibility, including the importance of engaging in health behaviors and participating in health decisions; (2) Health is doing what they want despite limitations; (3) Perceptions of health are shaped by personal factors (e.g., optimism, flexibility); and (4) Ratings/views of self-rated health are influenced by comparing themselves to others, a process serving to benchmark their health status. For these individuals, health was not abstract but linked to function, roles, strengths and learning (Whitmore et al., 2023).

Rajeski and Fanning (2019) note that most major contemporary models of health behavior change assume people value their health. If people value their health, it suggests they would be likely to engage in behaviors to enhance their health. An important and often unconsidered question is how individuals define their health (e.g., what does being healthy mean to the individual?) and whether those definitions are consistent with the health messages, interventions and outcomes that professionals advise. The analyses presented here describe the meaning of being healthy to a group of persons aging with a longstanding chronic condition (MS) and allow us to consider how health care that recognizes and incorporates these perceptions might be more effective.

## 3 | METHODS

Data for these analyses were collected as part of an ongoing longitudinal study following a sample of community residing persons with physician-diagnosed MS over a 28-year period. Details about the initial recruitment of the sample and data collection procedures are provided in earlier publications (Stuifbergen et al., 2000, 2016). A core battery of instruments was completed each year and selected measures were completed only at specific time points For example, the revised-Health Conception Scale (Lusk et al., 1995) was included in the Year 1 survey and the Year 26 survey to assess the meaning of health and how perspectives had changed over time. All aspects of this study were reviewed and approved by The University of Texas at Austin Institutional Review Board. Data reported in these analyses reflect responses to the original survey completed in 1996 (Time 1) and from the survey completed in 2022 (Time 26).

### 3.1 | Data collection procedures

Survey questionnaires have been sent annually to those who enrolled in the longitudinal study unless participants specifically requested to withdraw from the study, had died, or were lost to follow-up. As expected, the number of participants enrolled in the study has declined over time due to death, inability or lack of interest to continue in the study and loss to follow-up. Survey response rates at each time point have ranged from 66.0% to 89.6% of eligible respondents. At Time 26, 253 persons (41%) of the original 621 recruited into the longitudinal component remained in the study and were mailed an annual questionnaire. A total of 168 (70%) of the 253 persons returned their survey at Time 26.

### 3.2 | Measures

Table 1 provides information on the mean, standard deviation, and internal consistency reliability of the measures used in this analysis.

The revised version of Laffrey’s (1986) Health Conception Scale (HCS—R) (Lusk et al., 1995) was used to assess what “health” or “being healthy” means to individuals. This 16-item scale asks respondents to indicate their degree of agreement on a 6-point scale (1 = *strongly disagree* to 6 = *strongly agree*). Item scores are summed into two subscales: a 7-item clinical subscale with statements reflecting a more biomedical or clinical definition of health as absence of disease/illness and a 9-item wellness subscale representing health in more functional and adaptive terms. In our sample, the internal consistency reliability for the two subscales was strong with Cronbach’s alpha values at Times 1 and 26, respectively, of 0.90 and 0.88 for the Clinical subscale and 0.84 and 0.86 for the Wellness subscale. We conducted an exploratory factor analysis and found the principal component factor analysis generally confirmed the results of previous analyses positing two factors: clinical definition and wellness-oriented definition.

Participants were asked to respond to a single item rating their overall health at the present time on a 4-point scale with ratings of (1) poor, (2) fair, (3) good, and (4) excellent. Health-Promoting Behaviors were operationalized with the 52-item Health-Promoting Lifestyle Profile II (HPLPII), a scale that asks respondents to report the frequency they engage in a variety of activities directed toward increasing their health and well-being (Walker et al., 1995) using a response scale from 1 (*never*) to 4 (*routinely*). Responses to the HPLP-II items are summed into six subscales: Physical Activity, Spiritual Growth, Health Responsibility, Interpersonal Relations, Nutrition, and Stress Management. Evidence supports the use of the HPLP as a valid and reliable measure of health behavior in persons with MS (Stuifbergen, 1995).

Outcome measures varied at Times 1 and 26 of the study. At Time 1, respondents completed the Quality of Life Index—MS Version (Ferrans & Powers, 1992), a 72 item instrument measuring the individual’s satisfaction with and the relative importance of various quality of life domains. At Time 26, outcome measures included a one-item measure asking them to rate their “overall quality of life” from 1 = *very poor* to 10 = *very good* and the Physical and Emotional Role Functioning and Social Functioning Scales of the widely used and validated MOS SF36 (Ware & Sherbourne, 1992). The SF-36 is a multi-domain scale that includes items that measure role limitations due to physical health problems (4 items), social functioning (2 items), and role limitations due to emotional problems (3 items). Rating scales were coded and recalibrated following standard scoring procedures to a 0 (*least favorable*) to 100 (*most favorable*) scale.

### 3.3 | Data analysis

All surveys were proofed for complete data and entered into data files for analysis with SPSS version 27. All data were checked for out-of-range values and a random sample checked for accuracy of data entry. Descriptive statistics (means, standard deviations) were used to describe the sample and instrument responses. Because some distributions were not normally distributed and perceived health (self-rated health) was an ordinal measure, we chose to report and interpret the non-parametric statistics. Wilcoxon Signed Ranks were used to compare scores at the two time points and Spearman rho correlations were used to explore relationships between the Health Conception Scale scores, self-rated health and other variables. The sample size of 168 was adequate for detecting a small effect size of paired group differences (*r* = 0.25) and weak associations (*r* = 0.25) at a significance level of 0.05 and a power of 0.80, as determined by G*Power 3.1 (Faul et al., 2007).

## 4 | RESULTS

The sample used for the analyses described below included the 168 persons with MS who had returned the survey at Time 1 (1996) and again at Time 26 (2022). In 2022, the participants ranged in age from 48 to 94 (mean = 70.13, *SD* = 8.19) and had been diagnosed with MS for an average of 34.47 years (*SD* = 6.27). By contrast, the average age of the sample was 43.80 in 1996 and they had been diagnosed with MS for an average of 8.47 years. The majority of participants were female (90%), white/non-Hispanic (96%), and married (63%). In general, the study participants were well-educated as 43% had completed high school and 44% had completed college (bachelor’s or graduate degrees). Given the age of the sample it is not surprising that only 13% were currently employed either full or part-time.

There were many statistically significant differences between the sample of longitudinal “survivors” for this study (*n* = 168; those who completed Times 1 and 26) and those who were in the study at Time 1 (*n* = 654) but had dropped out some time before Time 26. However, only 3 of the differences between the groups at baseline reached a medium effect size (Cohen’s *d* = 0.50 or greater). At baseline (Time 1), those in the group “surviving” to Time 26 were significantly younger (*d* = 0.50), had fewer functional limitations (*d* = 0.70) and engaged in more physical activity (*d* = 0.57) than those who did not remain in the sample at Time 26.

The mean scores for each item of the Health Conception Scale-R at Times 1 and 26 are presented in Table 2. In addition, the average item score for the Clinical and Wellness subscales are presented. Given the different number of items on the two subscales, average items scores were used to compare the two subscales. The average item scores on the wellness subscale are higher than the average item clinical scores at both Times 1 and 26, indicating that respondents had stronger agreement with items describing a more functional and adaptive meaning of health. Subscale scores decreased significantly across the 26-year time period. The Wilcoxon signed-rank tests revealed that the median of the Clinical subscale at Time 26 (median = 29.00) was significantly lower than the median at Time 1 (median = 33.00, *Z* = −2.49, *p* = 0.013). The same result was found in the Wellness subscale between Time 1 (median = 46.00) and Time 26 (median = 43.44, *Z* = −3.56, *p* < 0.001). The effect sizes for the differences in the Clinical subscale (*r* = −0.19) and in the Wellness subscale (*r* = −0.27) were small.

As seen in Table 2, mean scores on each of the individual items also decreased from Times 1 to 26. The greatest decreases were in item 11 “Being Healthy means I do not require medications”; Item 16—“Being Healthy means my mind and body function at their highest level”; and Item 9—“Being Healthy means actualizing my highest and best aspirations.”

Interestingly, the correlation between the two subscale scores (Clinical and Wellness) were stronger at each time point (*r*_s_ = 0.41, *p* < 0.001 at Time 1 and *r*_s_ = 0.45, *p* < 0.001 at Time 26) than the correlations across time for each subscale. The Times 1 and 26 Clinical scores were correlated at *r*_s_ = 0.25, *p* < 0.01 and the Wellness Times 1 and 26 scores were correlated at *r*_s_ = 0.28. *p* < 0.001. There were no significant relationships between either the clinical or wellness subscale scores with age, years diagnosed or years of education at Time 1. At time 26, the clinical scale had a small correlation (*r*_s_ = 0.18, *p* < 0.05) with years diagnosed with MS and the Wellness subscale had a small (*r*_s_ = 0.16, *p* < 0.05) correlation with years of education.

Respondents were asked to indicate how they rated their overall health—poor, fair, good or excellent at both time points. The Health Self Rating Scale scores decreased slightly from Time 1 (mean = 2.73, *SD* = 0.75) to Time 26 (mean = 2.66, *SD* = 0.69) but there was no significant difference in perceptions of health status over time (*Z* = −1.04, *p* = 0.301). As seen in Figure 1, there were modest changes in the distribution of the responses as more respondents rated their health as “fair” at Time 26 (38%) compared to Time 1 (27%). At Time 1, 67% of the respondents rated their overall health as “good” or “excellent” and only 60% gave similar ratings at Time 26.

As seen in Tables 3 and 4, we also explored the relationships among HCS-R and Self-Rated Health scores to the total and subscale scores of the Health Promoting Lifestyle Profile-II (HPLP-II) at both time points. At Time 1, there were no significant relationships between the HPLP-II total score and the clinical or wellness subscale. However, there were a few weak but significant correlations between the HPLP II subscale scores and the clinical and wellness scores. Higher scores (greater agreement) with the clinical definition of health were negatively associated with the health responsibility subscale (*r*_s_ = −0.21, *p* < 0.01) and the interpersonal relationships subscale (*r*_s_ = −0.20, *p* < 0.05). Higher scores on the wellness subscale were positively associated with scores on the spiritual growth subscale of the HPLP-II (*r*_s_ = 0.17, *p* < 0.05).

At Year 26, the relationships between definitions of health and health behaviors were somewhat stronger. The total HPLP-II score had a small negative correlation with the clinical subscale score (*r*_s_ = −0.18, *p* < 0.05) and a small positive correlation with the wellness subscale (*r*_s_ = 0.24, *p* < 0.01) indicating that a more biomedical definition of health was associated with reports of less frequent health behaviors and stronger agreement with functional and adaptive meanings of health was associated with more frequent health behaviors. Only one of the six HPLP-II subscales—health responsibility—was significantly correlated with the clinical definition of health at Time 26 (*r*_s_ = −0.22, *p* < 0.01). Five of the six HPLP-II domain scores were significantly related to scores on the wellness domain (Health Responsibility *r*_s_ = 0.17, *p* < 0.05; Nutrition *r*_s_ = 0.18, *p* < 0.05; Spiritual Growth *r*_s_ = 0.31, *p* < 0.001; Interpersonal Relationships *r*_s_ = 0.24, *p* < 0.01; and Stress management *r*_s_ = 0.21, *p* < 0.01). The score on the one-item measure of perceived overall health was significantly related to total scores on the HPLP-II at Time 1 (*r*_s_ = 0.45, *p* < 0.001) and Time 26 (*r*_s_ = 0.45, *p* < 0.001).

With regard to relationships between the HCS-R and Self-Rated Health scores with the outcome variables, at Time 1 scores on the Quality of Life Index were negatively related to agreement with a clinical definition of health (*r*_s_ = −0.21, *p* < 0.01) and there was a strong positive relationship (*r*_s_ = 0.54, *p* < 0.001) between self-rated health and the quality of life index scores.

At Year 26, health self-ratings had strong positive relationships with all outcome scores. Scores on the HSR and one-item QOL measure were strongly related (*r*_s_ = 0.61, *p* < 0.001) as well as the Role Physical score (*r*_s_ = 0.46, *p* < 0.001), Role Emotional (*r*_s_ = 0.43, *p* < 0.001), and Social Role functioning scores (*r*_s_ = 0.44, *p* < 0.001). There were no significant relationships between scores on the clinical subscale of the HSC-R and the outcomes at Time 26, but HSC-R wellness scores had small significant positive correlations with overall quality of life (*r*_s_ = 0.21, *p* < 0.01) and the Role-Physical Functioning score (*r*_s_ = 0.15, *p* < 0.05).

## 5 | DISCUSSION

This study sought to identify perceptions of health held by persons aging with a chronic disabling condition and to explore how these perceptions changed over time. Understanding how people aging with a chronic condition view their health is essential for planning and delivering person-centered care, especially the delivery of health messages. Consistent with Whitmore et al. (2023), the majority (60%) of participants rated their overall health as good or excellent despite their average age and their experience of living with a chronic disabling neurological condition for more than 25 years. While ratings of overall health had decreased over time, the difference was not significant. It is important for health care providers who have been educated and trained clinically in an illness-care system to understand that even with aging and a limiting chronic condition, many participants still view their overall health as positive (Krahn et al., 2021). Providers can build on these positive health perceptions to encourage their patients to engage in positive health practices that will continue to enhance their health.

Findings related to the Revised Health Conception Scale (Lusk et al., 1995) were consistent with the literature supporting multidimensional views of health (Krahn et al., 2021; Van Druten et al., 2022). Participants had relatively high levels of agreement with all items on the scale and there was a moderate correlation between clinical and wellness subscale scores at both time points. Participants consistently agreed more strongly with items reflecting a wellness-oriented view of health than those reflecting a more biomedical model of health.

Scores on both subscales of the Health Conception Scale-R changed significantly over time with lower levels of agreement at Time 26 than Time 1. This may reflect the impact of aging and overall life experience on one’s perceptions of what being healthy means as the participants’ average age increased from 44 years at Time 1 to 70 years at Time 26. This is supported by the small (*r*_s_ = 0.26, *r*_s_ = 0.28) significant correlations between Time 1 and Time 26 subscale scores for clinical and wellness respectively. It is also possible that there may be other important aspects of being healthy for persons living with a chronic disabling condition that were not assessed in this scale. For example, Whitmore et al.’s (2023) participants emphasized the importance of health as a responsibility, an aspect not addressed in the HCS-R. Clearly, being healthy can mean something different to each individual at different times and in different contexts. Health care providers cannot assume that demographic or illness conditions are predictive for how individuals view being healthy. To plan and deliver effective person-centered care, health care providers should explore conceptions of health in dialogue with their patients (Van Druten et al., 2022).

A second area of inquiry addressed the relationships among definitions of health, self-rated health and other health-related variables and outcomes as well as changes over the 26-year time period. It should be noted that many of the significant correlations were weak, less than 0.30. The relatively weak explanatory power of many correlations limits the clinical significance of these individual findings. However, the consistent pattern of correlations at Times 1 and 26 suggests that while the relationships are small, this variance may be uniquely important. Overall, scores reflecting stronger agreement with a clinical definition of health were negatively related to reported frequency of health behaviors and stronger agreement with a wellness definition of health were positively related to reports of health behaviors such as spiritual growth and interpersonal relationships at Year 26. Our findings, like those of Gessert and colleagues (2015) with rural samples, suggest that persons aging with chronic conditions might be more responsive to health messages that emphasize function in daily living, carrying out normal responsibilities, and adjusting to changes in environment and demands. Since quality-of-life outcome measures were different at Times 1 and 26, no comparison of relationships was possible.

Findings from this study must be interpreted cautiously as they rely on responses from a unique sample of persons who are long-term ‘survivors’ in a longitudinal study. As noted, this sample of “survivors” in a longitudinal study differed significantly at baseline from the larger group of persons with MS that was no longer participating in the study. In addition to survivors being significantly younger and less impaired at baseline than those no longer in the study, it is also possible that those who continued to persist and participate over such a long period of time had a slower trajectory of MS-related impairment. It is likely that persons in this sample are more interested in health and their ability (resources, limited limitations, support) to maintain participation in the study over such an extended time is likely different than the broader population of persons with MS. In addition, the length of follow-up (26 years) accentuated a common issue experienced with all longitudinal studies, the attrition of many participants. While response rate throughout the many years of the study were consistently high from actively enrolled participants, many of the original participants had been lost due to death, inability to continue in the study due to increasing impairment and the research team’s inability to deliver the survey to a current address.

The original participants were recruited for a cross sectional study and varied in age and length of diagnosis at the initiation of the study. Thus, while changes in views of health and other variables reflect the passage of time, these changes cannot be specifically anchored to time since diagnosis or a specific age. Furthermore, the major changes in overall societal views and advances in diagnosis and medical treatment of MS over this extended time period may have influenced scores on all measures in unknown ways. The well-known limitations associated with use of self-reports must be balanced with the feasibility of examining these constructs in a community-residing sample from both rural and urban settings in a large southwestern state.

In conclusion, overall, findings suggest that the meaning of health for individuals aging and living with a chronic disabling condition is complex and subject to change over time. The strong agreement with items reflecting an adaptive and functional view of health should be considered by health care professionals when designing interventions and health messages.

## Figures and Tables

**FI GURE 1:**
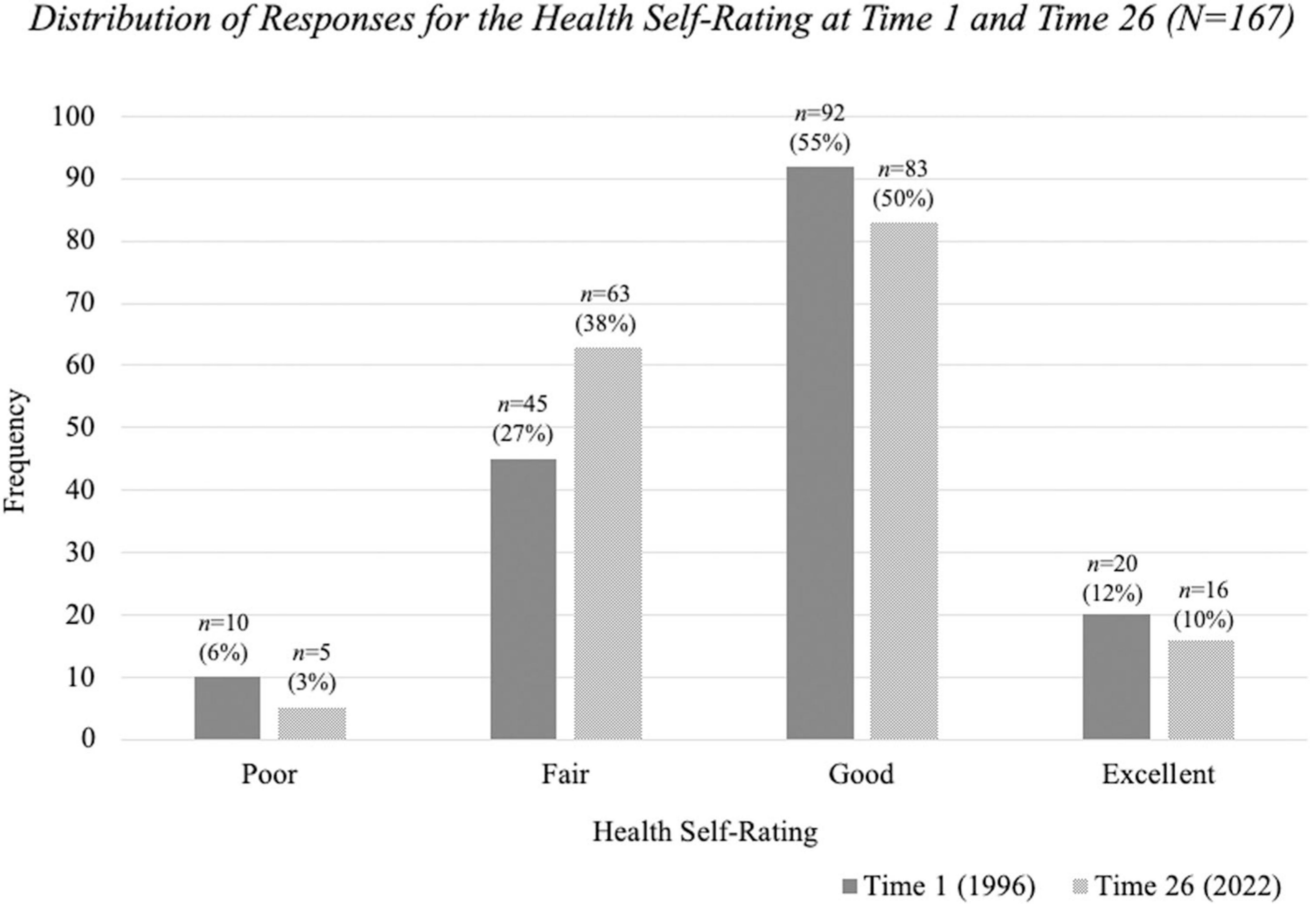
Distribution of responses for the health self-rating at Times 1 and 26 (*N* = 167).

**TABLE 1:** Mean, standard deviation, Cronbach’s alpha of the measures in Times 1 and 26 (*N* = 168).

	Time 1 (1996)			Time 26 (2022)		
		
Measures	*M*	*SD*	Cronbach’s *α*	*M*	*SD*	Cronbach’s *α*
HCS-R						
Clinical	30.91	8.69	0.90	28.98^a^	8.52	0.88
Wellness	44.81	6.96	0.84	42.52	7.34	0.86
HSR (4-point Likert)	2.73	0.75	-	2.66^a^	0.69	-
HPLP-II						
Health responsibility	22.82^a^	5.06	0.77	24.29	5.15	0.82
Physical activity	17.40^a^	5.68	0.81	17.27	6.34	0.88
Nutrition	23.96^a^	4.82	0.69	25.07	5.09	0.77
Spiritual growth	28.43^a^	5.13	0.85	28.62	5.18	0.88
Interpersonal relations	28.90^a^	4.32	0.77	28.18	4.75	0.83
Stress management	21.41^a^	4.37	0.74	23.51	4.40	0.76
Total scores	142.92^a^	20.17	0.91	146.94	23.16	0.94
Quality of life						
Quality of Life Index—MS Version	21.37	4.24	0.92	-	-	-
Quality of life (1 item)	-	-	-	7.53^a^	1.83	-
MOS SF-36 (transformed scores)						
Role physical	-	-	-	53.94	29.36	0.94
Role emotional	-	-	-	71.03	29.28	0.94
Social functioning	-	-	-	66.89	29.02	0.80

*Note*: *M* = mean; *SD* = standard deviation.

Abbreviations: HCS-R, the revised Health Conception Scale; HSR, Health Self-Rating; HPLP-II, Health Promoting Lifestyle Profile II; MOS SF-36, Medical Outcomes Study 36-item short form.

a *N* = 167.

**TABLE 2:** Item and subscale scores for the Clinical and Wellness Subscales on the revised Health Conception Scale (*N* = 168).

	Time 1 (1996)				Time 26 (2022)				Possible range	
			
Subscales	Item *M*	*SD*	Subscale *M*	*SD*	Item *M*	*SD*	Subscale *M*	*SD*	Item *M*	Subscale *M*
Clinical conception	4.42	1.24	30.91	8.69	4.14	0.82	28.98^a^	8.52	1–6	7–42
Wellness conception	4.98	0.77	44.81	6.96	4.72	0.82	42.52	7.34	1–6	9–54
Item						Time 1 (1996)		Time 26 (2022)		Possible range
	
						Item *M*	*SD*	Item *M*	*SD*	
Clinical conception										
2. Being free from symptoms of disease						4.79	1.35	4.31	1.58	1–6
3. Not requiring a doctor’s services						4.37	1.59	4.26	1.54	1–6
5. Not requiring pills for illness or disease						4.04	1.68	3.95^a^	1.66	1–6
6. Not being under a doctor’s care for illness						4.02	1.71	3.93	1.65	1–6
8. Not being sick						5.05	1.36	4.83^a^	1.27	1–6
11. I do not require medications						4.03	1.76	3.45	1.88	1–6
15. Having no physical or mental incapacities						4.62	1.59	4.20	1.63	1–6
Wellness conception										
1. Feeling great—on top of the world						4.66	1.25	4.27	1.41	1–6
4. Adjusting to life’s changes						4.94	1.23	4.83	1.10	1–6
7. Being able to change and adjust to demands made by the environment						4.96	1.18	4.74	1.11	1–6
9. Actualizing my highest and best aspirations						4.84	1.24	4.44	1.37	1–6
10. Adequately carrying out my daily responsibilities						5.14	1.05	4.91	1.05	1–6
12. Carrying on the normal functions of daily living						5.18	0.97	4.96	1.02	1–6
13. Coping with changes in my surroundings						5.01	1.15	4.81	1.14	1–6
14. Fulfilling my responsibilities as a husband/wife/daughter/friend/worker etc.						5.02	1.27	4.94	1.04	1–6
16. My mind and body function at their highest level						5.05	1.24	4.62	1.35	1–6

*Note*: *M* = mean; *SD* = standard deviation.

a *N* = 167.

**TABLE 3:** Spearman’s Rho correlation matrix of the study variables at Time 1 (*N* = 168).

	1	2	3	4	5	6	7	8	9	10
1. HCS-R–Clinical	-									
2. HCS-R–Wellness	0.41***	-								
3. HSR	−0.07	0.06	-							
4. HPLP-II–Health Responsibility	−0.21**^a^	0.07^a^	0.03^a^	-						
5. HPLP-II–Physical Activity	−0.08^a^	0.13^a^	0.31***^a^	0.26***^a^	-					
6. HPLP-II–Nutrition	0.07^a^	0.10^a^	0.19*^a^	0.35***^a^	0.34***^a^	-				
7. HPLP-II–Spiritual Growth	−0.12^a^	0.17*^a^	0.23**^a^	0.47***^a^	0.23**^a^	0.36***^a^	-			
8. HPLP-II–Interpersonal Relations	−0.20*^a^	0.11^a^	0.16*^a^	0.52***^a^	0.18*^a^	0.28***^a^	0.70***^a^	-		
9. HPLP-II–Stress Management	−0.10^a^	0.06^a^	0.18*^a^	0.39***^a^	0.28***^a^	0.30***^a^	0.46***^a^	0.46***^a^	-	
10. HPLP-II–Total Scores	−0.14^a^	0.15^a^	0.27***^a^	0.72***^a^	0.57***^a^	0.64***^a^	0.76***^a^	0.74***^a^	0.68***^a^	-
11. QoL Index	−0.21**	−0.04	0.54***	0.18*^a^	0.31***^a^	0.24**^a^	0.49***^a^	0.41***^a^	0.29***^a^	0.45***

*Note*:

* *p* < 0.05

** *p* < 0.01

*** *p* < 0.001.

Abbreviations: HCS-R, the revised Health Conception Scale; HSR, Health Self-Rating; HPLP-II, Health Promoting Lifestyle Profile II; QoL Index, Quality of Life Index–MS Version.

a *N* = 167.

**TABLE 4:** Spearman’s Rho correlation matrix of the study variables at Time 26 (*N* = 168).

	1	2	3	4	5	6	7	8	9	10	11	12	13
1. HCS-R–Clinical	-												
2. HCS-R–Wellness	0.45***^a^	-											
3. HSR	−0.03^b^	0.21**^a^	-										
4. HPLP-II–Health Responsibility	−0.22**^a^	0.17*	0.22**^a^	-									
5. HPLP-II–Physical Activity	−0.12^a^	0.08	0.32***^a^	0.38***	-								
6. HPLP-II–Nutrition	−0.09^a^	0.18*	0.28***^a^	0.50***	0.41***	-							
7. HPLP-II–Spiritual Growth	−0.12^a^	0.31***	0.46***^a^	0.47***	0.31***	0.45***	-						
8. HPLP-II–Interpersonal Relations	−0.12^a^	0.24**	0.45***^a^	0.56***	0.34***	0.52***	0.75***	-					
9. HPLP-II–Stress Management	−0.12^a^	0.21**	0.35***^a^	0.57***	0.37***	0.42***	0.68***	0.60***	-				
10. HPLP-II–Total Scores	−0.18*^a^	0.24**	0.45***^a^	0.77***	0.65***	0.72***	0.79***	0.81***	0.78***	-			
11. QoL (1 item)	−0.01^b^	0.21**^a^	0.61***^a^	0.19*^a^	0.30***^a^	0.30***^a^	0.50***^a^	0.50***^a^	0.34***^a^	0.47***^a^	-		
12. MOS SF-36 Role Physical	0.07^a^	0.15*	0.46***^a^	0.13	0.36***	0.27***	0.28***	0.28***	0.18*	0.32***	0.47***^a^	-	
13. MOS SF-36 Role Emotional	−0.11^a^	0.08	0.43***^a^	0.13	0.22**	0.17*	0.45***	0.34***	0.30***	0.35***	0.46***^a^	0.59***	-
14. MOS SF-36 Social Functioning	−0.09^a^	0.14	0.44***^a^	0.14	0.29***	0.32***	0.39***	0.39***	0.21**	0.38***	0.61***^a^	0.63***	0.62***

*Note*:

* *p* < 0.05

** *p* < 0.01

*** *p* < 0.001.

Abbreviations: HCS-R, the revised Health Conception Scale; HSR, Health Self-Rating; HPLP-II, Health Promoting Lifestyle Profile II; MOS SF-36, Medical Outcomes Study 36-item short form; QoL, Quality of Life.

a *N* = 167.

b *N* = 166.

## Data Availability

The data that support the findings of this study are available from the corresponding author upon reasonable request.
